# Metal-Ion Coordination Self-Assembled Green Synthesized Gold Nanoparticles for 4‑Nitrophenol Reduction and Food Colorants Degradation

**DOI:** 10.1021/acsomega.5c06971

**Published:** 2025-09-22

**Authors:** Josue Abraham Lara Zavala, Edgar Iván Ocampo López, Mora Fonseca Mónica Cecilia, Helena Cortés Reyes, Eduardo Silva Beltrán, Naveen Kumar Reddy Bogireddy

**Affiliations:** Instituto de Ciencias Físicas, National Autonomous University of Mexico (UNAM), Mexico C.P. 04510, Mexico

## Abstract

The field of responsive nanometric systems is rapidly expanding, offering significant potential for environmental and catalytic applications. In this study, we report the green synthesis of metal ion-directed self-assembled gold nanoparticles (AuNPs) mediated by *Pithecellobium dulce* (*P. dulce*) leaf extracts. This biosynthetic approach facilitates dynamic nanoparticle aggregation through metal-ion coordination, enhancing surface chemistry interactions and core nanoparticle organization. The precise molecular mechanism governing the self-assembly process remains to be fully elucidated. Nevertheless, these nanostructures (∼12 nm, face-centered cubic) exhibit remarkable catalytic activity, achieving near-complete reduction of 4-nitrophenol within 25 min and efficient degradation of azo food dyes, such as Tartrazine, Allura Red AC, and Brilliant Blue FCF, with removal efficiencies of up to 98%. Characterization by UV–visible, TEM, XPS and FTIR confirmed nanoparticle formation, biofunctionalization, and the potential interaction with target molecules. These results highlight the dual functionality of these green-synthesized AuNPs. They demonstrate their potential as versatile and environmentally friendly catalysts, as well as their high efficiency in water remediation, making them a promising solution to environmental challenges.

## Graphical abstract

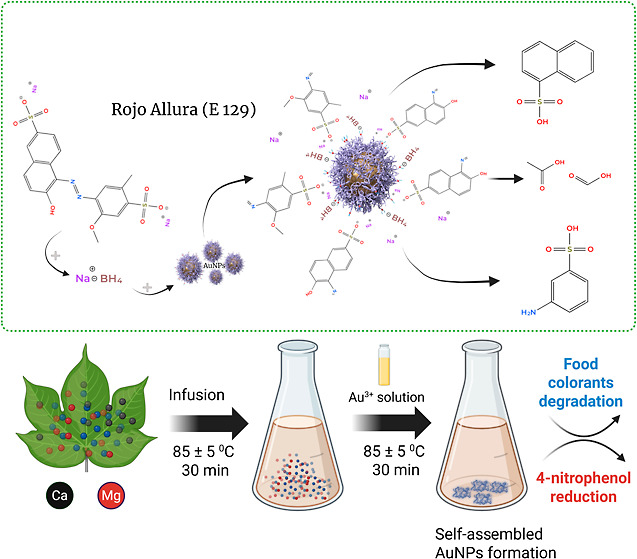

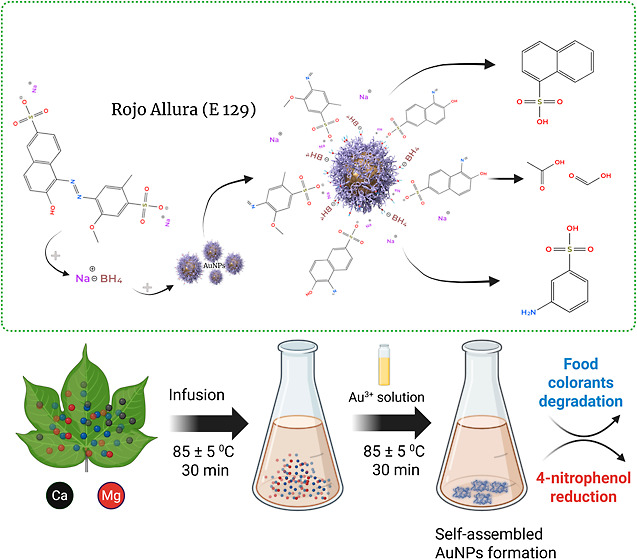

## 1 Introduction

Water pollution remains a critical global environmental challenge, necessitating the development of effective and sustainable remediation strategies.^1–3^ The diversity of pollutants varying in size, chemical composition, persistence, and toxicity reflects the complexity of this problem. Pollutants are commonly classified according to size into macro, micro, and nanoscopic species.^4,5^ Macro-pollutants, such as plastic debris and other solid waste larger than 5 mm, predominantly originate from anthropogenic activities and can be removed through conventional remediation techniques.^6^ In contrast, smaller-scale contaminants present a more significant challenge due to their persistence, bioavailability, and limited removability by standard treatment methods. These include suspended particulate matter (SPM) such as PM10, PM2.5, and ultrafine particles (PM1 and PM0.1), which pose well-documented risks to human health and ecosystems due to their capacity to penetrate biological barriers and induce oxidative stress.^7,8^

Molecular pollutants, such as 4-nitrophenol (4-NP) and synthetic azo dyes, widely used in the food industry, represent another class of emerging contaminants.^9,10^ Food-grade dyes such as Brilliant Blue FCF (E133), Allura Red AC (E129), and Tartrazine (E102) are extensively employed as colorants in processed foods across several countries,^11^ including Mexico, where they are frequently found in beverages, candies, and other ultraprocessed products targeted at children and young people. This widespread use highlights the importance of assessing potential health risks.^12–16^ Among these, Allura Red AC has attracted toxicological attention for its association with hyperactivity in children, allergic reactions, and emerging evidence of genotoxic and pro-inflammatory effects.^13,17–21^ Although regulatory agencies have established acceptable daily intake (ADI) limits (6–10 mg/kg depending on the dye), concerns remain regarding widespread consumption in vulnerable populations.^22–26^

Similarly, 4-NP, a pale-yellow aromatic compound, is widely used as an intermediate in the production of dyes, pesticides, pharmaceuticals, and fungicides,^27,28^ and as a model compound in studies of catalysis and environmental remediation.^29–34^ Its presence in aquatic environments results from industrial discharge and the degradation of organophosphate pesticides, such as parathion and methyl parathion.^26,35–37^ Exposure to 4-NP can cause diverse toxic effects, from nausea and headaches at low doses to hepatotoxicity and methemoglobinemia at higher exposures.^38,39^ Its high-water solubility, chemical stability, and bioaccumulative potential make it an ecotoxicological concern, especially in agricultural regions.^33^ Despite these risks, regulatory frameworks for 4-NP remain limited or nonspecific in many jurisdictions.

Given these challenges, the development of efficient, selective, and environmentally benign strategies for degrading persistent organic pollutants has become a research priority.^40^ In this context, AuNPs synthesized via green chemistry approaches have gained considerable interest due to their exceptional catalytic activity, high surface-to-volume ratio, and biocompatibility.^41–45^ Plant infusion provides natural reducing and stabilizing agents, including flavonoids, polyphenols, and sugars, which influence nanoparticle formation, self-assembly, and functional properties.^46–51^ *Pithecellobium dulce* (guamúchil) leaves represent a promising, underutilized natural resource rich in bioactive compounds^52–54^ capable of directing the formation and stabilization of AuNPs. However, their application in nanoparticle-mediated degradation of molecular pollutants remains underexplored.

In this study, we synthesized AuNPs using an aqueous infusion of *P. dulce* leaves and evaluated their catalytic performance in the reduction of 4-NP and the degradation of selected synthetic food dyes in aqueous media. This biosynthetic strategy aligns with green chemistry principles, offering a scalable, low-cost, and eco-friendly alternative to conventional chemical synthesis. We further investigated the role of phytochemical-mediated self-assembly in the formation of hierarchical nanostructures and its influence on catalytic efficiency. Our results contribute to advancing plant-based nanotechnologies for environmental remediation, highlighting the dual function of *P. dulce* infusion as both reducing agents and supramolecular directors in green nanoparticle synthesis, with promising applications for the removal of persistent organic contaminants from aquatic environments.

## 2 Experimental Section

### 2.1 Materials and Methods

Chloroauric acid (HAuCl_4_·3H_2_O, ≥99.9%), 4-nitrophenol (4-NP, ≥99.5%), and sodium borohydride (NaBH_4_, ≥98%) were obtained from Sigma-Aldrich and used without further purification. Fresh leaves of *P. dulce* were collected from mature trees located on the campus of the Autonomous University of the State of Morelos (UAEM), Mexico. The leaves were thoroughly rinsed with distilled water to eliminate surface debris and air-dried at room temperature prior to their use in the synthesis.

### 2.2 Preparation of the *P. dulce* Leaves Infusion

The preparation of the infusion from fresh *P. dulce*leaves was carried out to ensure the purity and consistency of the infusion. Fresh leaves were thoroughly rinsed with tap water followed by distilled water to remove surface impurities and subsequently air-dried at room temperature. A total of 15 g of dried leaves was weighed per batch. Each leaf was manually cut into four smaller sections and added to 60 mL of deionized water in a glass beaker. The mixture was heated to 70 °C for 30 min under constant supervision. After heating, the infusion was allowed to cool to room temperature and then filtered by gravity using Whatman filter paper (pore size: 0.2 μm) to remove residual solids. The resulting filtrate, rich in phytochemicals and naturally occurring minerals such as calcium and magnesium,^55,56^ was used directly as the reducing and stabilizing agent in the green synthesis of AuNPs.

### 2.3 Synthesis of Gold Nanoparticles by Reduction Using *P. dulce* Infusion

The reduction of Au^3+^ ions to AuNPs were carried out using the freshly prepared *P. dulce* leaf infusion. In a typical synthesis, 5 mL of the infusion was mixed with 15 mL of deionized water in a glass beaker, resulting in a total volume of 20 mL. The mixture was heated to 60 °C and stirred continuously at 200 rpm for 30 min to activate the phytochemical constituents. Subsequently, 2 mL of a 10 mM aqueous solution of chloroauric acid (HAuCl_4_·3H_2_O) was added dropwise to the preheated infusion. The reaction mixture was then stirred for an additional 30 min under the same conditions. After this period, the beaker was removed from the heat source and allowed to cool naturally to room temperature without further agitation. Upon completion of the reaction, the solution exhibited a deep yellow color, characteristic of the *P. dulce* infusion. After refrigeration for 24 h, a distinct color changes from deep yellow to orange was observed, indicating the formation and stabilization of AuNPs. The as-prepared colloidal suspension was purified by three successive centrifugation–redispersion cycles with deionized water to remove unreacted HAuCl_4_ and excess soluble phytochemicals. A fraction of phytomolecules remained associated with the nanoparticle surface, acting as natural capping agents that maintain surface functionality of the AuNPs.

### 2.4 Optical and Structural Characterization of Gold Nanoparticles

The optical properties of the synthesized AuNPs were analyzed using UV–Vis spectroscopy. Absorbance spectra were recorded on a Hitachi UV-5100 spectrophotometer across the wavelength range relevant to the surface plasmon resonance (SPR) band characteristic of AuNPs. The structural characteristics of the synthesized AuNPs were investigated by X-ray diffraction (XRD) using a Bruker D8 Advance Eco diffractometer equipped with Cu Kα radiation (λ = 1.5406 Å). The diffraction patterns were used to identify crystalline phases and estimate the average crystallite size based on the Debye–Scherrer equation. High-resolution transmission electron microscopy (HR-TEM) and energy-dispersive X-ray spectroscopy (EDS) analyses were performed using a JEOL JEM-2200Fs microscope equipped with a spherical aberration corrector. HR-TEM provided detailed insights into particle size, morphology, and lattice fringes, while EDS confirmed the elemental composition of the nanoparticles. Fourier-transform infrared spectroscopy (FTIR) was conducted on a Nicolet iS10 FTIR spectrometer to identify the functional groups present in the *P. dulce* leaf infusion responsible for the reduction and stabilization of gold ions. The spectra were used to elucidate the interaction mechanisms between the phytochemical constituents and the nanoparticle surface. X-ray photoelectron spectroscopy (XPS) analysis, performed using a Physical Electronics PHI 5000 Versaprobe II equipped with an Al Kα source (1486.6 eV), enables the identification of elemental composition and chemical states, including the presence of metal ions associated with the AuNP surface.

### 2.5 4-Nitrophenol Reduction Assay

The catalytic efficiency of the AuNPs synthesized using *P. dulce* leaf infusion was evaluated through the model reduction of 4-NP to 4-aminophenol (4-AP). This well-established reaction is commonly employed to assess the catalytic activity of noble metal nanoparticles and is visually indicated by a distinct color change from deep yellow (4-NP) to colorless (4-AP). For each assay, the reaction mixture was prepared by combining 1 mL of deionized water, 100 μL of a 1 mM aqueous solution of 4-NP, and 100 μL of a freshly prepared 100 mM sodium borohydride (NaBH_4_) solution. The reaction was initiated by the addition of either 5 or 10 μL of the AuNP colloidal suspension. The progress of the reaction was monitored spectrophotometrically by measuring the time-dependent decrease in the absorbance of the characteristic peak of 4-NP at ∼400 nm, allowing for the evaluation of the catalytic performance of the biogenic AuNPs.

### 2.6 Degradation of Azo Food Dyes

The catalytic efficiency of AuNPs synthesized using *P. dulce* leaf infusion was further evaluated in the reduction of synthetic food dyes commonly used in the Mexican food industry. The reactions were conducted under dark conditions to prevent photodegradation and ensure reproducibility, with reaction progress monitored by UV–visible spectrophotometry. The targeted dyes included commercially available formulations: yellow Tartrazine (E102), red Allura Red AC (E129), blue Brilliant Blue FCF (E133), and a green dye prepared as a mixture of Tartrazine (E102) and Brilliant Blue FCF (E133). These dyes were selected due to their widespread use and environmental relevance as persistent organic pollutants.

For each reduction experiment, the reaction mixture comprised 1 mL of deionized water, 100 μL of the dye solution, and 100 μL of a freshly prepared 100 mM aqueous sodium borohydride (NaBH_4_) solution. The catalytic process was initiated by adding 5 μL of the AuNP colloidal suspension synthesized from *P. dulce* infusion. Decolorization progress was tracked by monitoring the decrease in absorbance at the maximum absorption wavelength characteristic of each dye, enabling the assessment of the catalytic degradation efficiency and potential of the biogenic AuNPs for treatment of dye-contaminated aqueous media.

## 3 Results and Discussion

The structural and morphological characterization of the AuNPs synthesized using *P. dulce* infusion was conducted to elucidate their crystalline nature and nanoscale features. The X-ray diffraction (XRD) pattern of the AuNPs biosynthesized using *P. dulce* infusion, Figure 1a confirms their crystalline nature. The diffraction pattern exhibits characteristic peaks at 2θ values of 38.4°, 45°, 65°, and 77°, which correspond to the (111), (200), (220), and (311) crystallographic planes of face-centered cubic (FCC) gold, respectively. These results are consistent with the standard reference pattern provided by the Joint Committee on Powder Diffraction Standards (JCPDS No. 04-0784), where the most intense peak at 38.4° is assigned to the Au(111) plane. The average crystallite size of the nanoparticles was estimated using the Scherrer eq 1

1 D=(K×λ)/(β×cos(θ))

where λ = 1.5406 Å is the wavelength of Cu Kα radiation, β represents the full width at half-maximum (fwhm) of each peak (in radians), θ is the Bragg angle corresponding to half of the 2θ value, and *K* = 0.9 is the shape factor for roughly spherical particles. Applying this calculation to all four characteristic peaks ((111), (200), (220), and (311)) and averaging the resulting values yields an average crystallite size of approximately 12.7 nm (Figure S1). This value is in good agreement with the particle size determined by TEM (11.63 ± 0.46 nm), providing reassurance about the reliability of the results.

**Figure 1:**
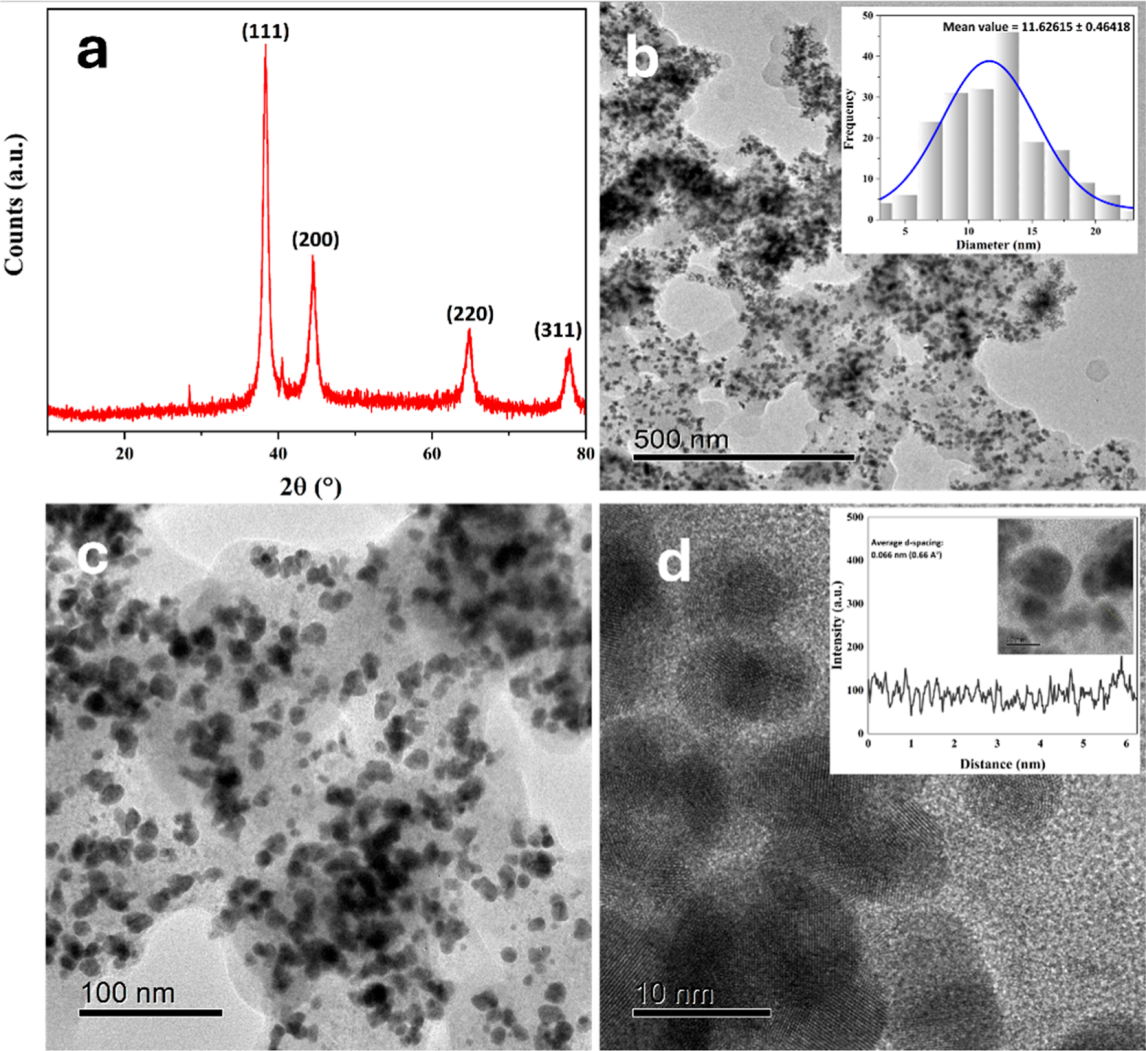
(a) X-ray diffraction (XRD) pattern of AuNPs synthesized using *P. dulce* infusion, showing characteristic gold peaks ((111), (200), (220), (311)). (b) HRTEM image at 500 nm scale showing well-dispersed, quasi-spherical nanoparticles; inset: particle size distribution (mean diameter 11.63 ± 0.46 nm). (c) HRTEM image at 100 nm scale illustrating ordered arrays of quasi-spherical nanoparticles. (d) HRTEM image at 10 nm scale showing closely packed nanoparticles. Interplanar spacing was determined from three HRTEM micrographs (Figure S1, Supporting Information), yielding average values of 0.34 nm ± 0.02, 0.28 nm ± 0.01, and 0.31 nm ± 0.02 nm, consistent with gold’s crystalline structure.

In contrast Figure 1b shows a transmission electron microscopy (TEM) image at 500 nm scale, revealing a broad and homogeneous distribution of nanoparticles. These nanoparticles display a pronounced tendency to self-assemble into ordered arrays, likely driven by supramolecular interactions mediated by the phytochemical components of the *P. dulce* infusion. The particle size distribution histogram included in the figure indicates a mean diameter of 11.63 ± 0.46 nm, confirming the nanometric scale and uniformity of the colloidal system.

At higher magnification, Figure 1c provides a TEM image at 100 nm scale, illustrating predominantly quasi-spherical nanoparticles with some polymorphism. The self-assembled structures are more clearly defined at this resolution, underscoring the influence of noncovalent forces and the phytochemical matrix on the spatial organization of the particles. These TEM micrographs also reveal the tendency of the nanoparticles to form dense clusters and branched arrangements, indicative of controlled, nonrandom aggregation into submicrometric hierarchical networks. At higher magnification, the formation of interconnected chains and close-packed structures with well-defined edges becomes evident, highlighting a directional self-assembly behavior. This biodirected assembly, driven by phytochemicals in the *P. dulce* infusion, not only governs the spatial organization but also potentially enhances functional properties such as plasmonic coupling and catalytic surface accessibility—features critical for environmental applications.

Furthermore, Figure 1d presents a TEM micrograph at a 10 nm scale, highlighting the fine structure of the self-assembled nanoparticles. The interplanar spacing was re-evaluated using three HRTEM micrographs (Figure S1), where regions with clear lattice fringes were selected and analyzed via FFT in ImageJ. The measured spacings were 0.34 ± 0.02 nm, 0.28 ± 0.01 nm, and 0.31 ± 0.02 nm, which are consistent with the expected crystalline structure of AuNPs. Variations are attributed to crystallographic orientation. The results confirm the presence of well-defined crystalline structures, indicative of a controlled synthesis and reliable structural characterization.

It is important to note that the minor differences between the crystallite size estimated from XRD (∼12.7 nm), and the average particle size obtained by TEM (11.63 ± 0.46 nm) are expected due to the fundamental differences between the techniques. The Scherrer equation estimates the size of coherent diffraction domains, which may include aggregated or polycrystalline regions. In contrast, TEM’s direct imaging capability allows it to directly visualize discrete, well-separated particles, providing a more accurate assessment of the actual nanoparticle size.

Building upon the structural and morphological characterization, energy-dispersive X-ray spectroscopy (EDS) analysis was performed to confirm the elemental composition of the synthesized AuNPs. The EDS spectra revealed a dominant gold content of 43.9 wt %, corroborating the formation of AuNPs. In addition, other elements such as carbon (26 wt %), oxygen (13.4 wt %), calcium (9.2 wt %), potassium (5.5 wt %), and magnesium (2 wt %) were detected on the nanoparticle surfaces.

These elements are likely derived from organic and inorganic compounds present in the *P. dulce* leaf infusion, which form a capping layer that stabilizes the nanoparticles. This phytochemical^52–54,57^ and mineral capping layer, further detailed in Table 1, is instrumental in modulating the surface reactivity of the AuNPs, thereby playing a significant role in their catalytic efficiency.

**Table 1:** Bioactive Compounds Reported in *P. dulce* ^52–54,57^

bioactive compounds reported in the leaves of P. dulce		
phytochemical name	molecular formula	molecular weight(g/mol)
octacosanol	C_28_H_58_O	410.8
α-spinasterol	C_29_H_48_O	412.7
kaempferol	C_15_H_10_O_6_	286.8
kaempferol-3-rhamnoside	C_21_H_19_O_10_	431.7
β-glucoside-α-spinasterol	C_35_H_58_O_6_	574.8
dulcitol	C_6_H_14_O_6_	182.2
quercetin	C_15_H_10_O_7_	302.2

These residual phytoconstituents are not merely byproducts but act as surface ligands that provide functional groups influencing electron transfer processes during catalysis. The detection of Mg and Ca in EDS mapping supports their origin from the *P. dulce* extract and their association with the AuNP surface. Although the nanoparticles were purified by repeated centrifugation with deionized water, a fraction of biomolecules remained strongly bound to the metallic core, a characteristic intrinsic to green synthesis routes. Their presence modulates the apparent catalytic kinetics, making these phytochemical capping agents integral to the overall functional performance of the AuNPs.

These results demonstrate that the green synthesis approach using *P. dulce* enables the production of AuNPs with functionalized surfaces, which can be advantageous for applications in catalysis, biomedicine, and pollutant remediation.

The mechanism of AuNPs formation mediated by *P. dulce* infusion is notably significant. Phytochemical constituents, particularly secondary metabolites such as polyphenols, flavonoids, and tannins, play a central role as reducing agents by converting Au^3+^ ions to metallic Au^0^, thereby initiating nucleation and subsequent nanoparticle growth. The detection of carbon and oxygen in the EDS analysis indicates that organic molecules from the infusion adsorb onto the nanoparticle surfaces, functioning as capping and stabilizing agents. During the initial reduction step, functional groups including hydroxyl (−OH) and carbonyl (−C=O) moieties act as electron donors to reduce gold ions. The adsorption of these biomolecules further modulates controlled nanoparticle growth. However, the interaction with metal cations such as Ca^2+^, K^+^, and Mg^2+^, identified through elemental analysis, adds complexity to the process. These metal cations coordinate with carboxylate groups, generating COO–M and COO–M^2+^–OOC moieties that neutralize the surface charge of the AuNPs. This intricate interaction influences the stabilization mechanism and may lead to aggregation or controlled precipitation of the nanoparticles.

Collectively, these findings suggest that *P. dulce* not only facilitates the reduction of gold ions but also forms a passivating metal ion coordination—organic biomass layer that may enhances the stability of the nanoparticles. This interaction profoundly affects the physicochemical and catalytic properties of the synthesized AuNPs, thereby contributing to their functional performance. The elemental composition and surface functionalization of the AuNPs are further illustrated in the EDS analysis presented in Figure 2.

**Figure 2:**
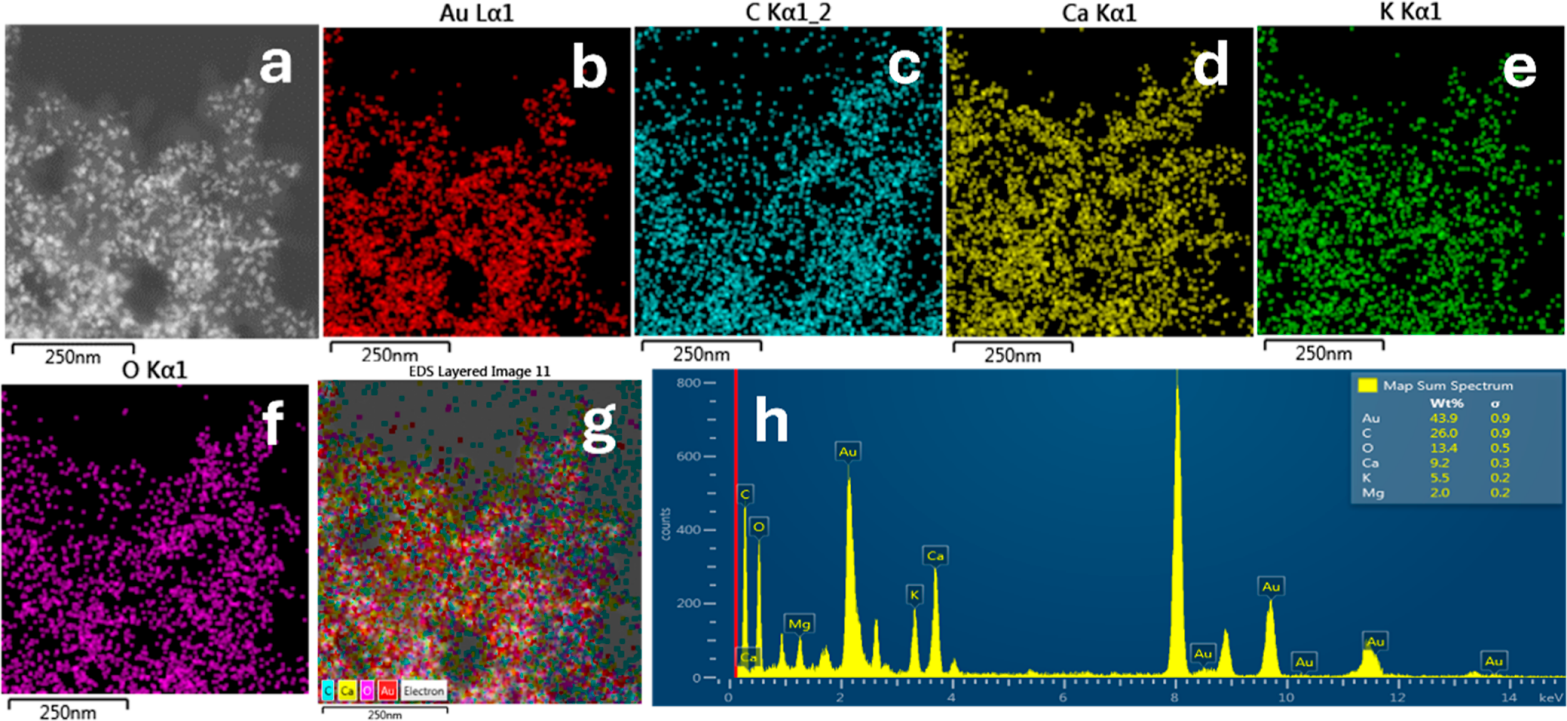
(a) Transmission electron microscopy (TEM) image of the AuNPs at 250 nm scale, showing well-dispersed, quasi-spherical particles. Elemental maps obtained by energy-dispersive X-ray spectroscopy (EDS) showing the distribution of (b) gold (Au), (c) carbon (C), (d) calcium (Ca), (e) potassium (K), and (f) oxygen (O), highlighting their spatial localization in and around the nanoparticles. Moreover, (g,h) present composite EDS mapping images, illustrating colocalization of multiple elements on the nanoparticle surfaces and confirming the presence of biomolecule capping and associated ions.

Complementing the EDS results, XPS has played a crucial role in confirming the presence of elemental and chemical states in AuNPs. The survey scan of AuNPs revealed major peaks at 82, 101, 283, 398, 530, and 1290 eV, corresponding to the characteristic peaks of Au 4f, Si 2p, C 1s, N 1s, O 1s, and Mg 1s, respectively. The detection of N 1s (398 eV) and Si 2p (101 eV) provides strong evidence for the incorporation of biomass species onto the nanoparticle surfaces. At the same time, trace amounts of nitrogen and silicon confirm the presence of residual organic constituents (Figure S2, Supporting Information). Deconvolution of the Au 4f peak further confirmed the chemical species present. It revealed a binding energy shift from 83.9 eV (for bare gold) to 82.8 eV, indicative of electron transfer interactions between AuNPs and Mg.^58^ This shift suggests partial electron donation from Mg^2+^ to the gold surface, modifying the electronic density of states and potentially enhancing catalytic sites.^59^

The XPS data, in conjunction with TEM-EDS, provides compelling evidence for the proposed coordination interactions between residual metal ions and the organic biomass capping layer. Such coordination likely alters the local chemical environment on the AuNP surface, stabilizing undercoordinated sites and modulating surface charge distribution. This has direct implications for the physicochemical behavior of the nanoparticles, as the modified electronic structure can influence adsorption energies of reactants, electron transfer rates, and overall catalytic kinetics. The presence of Mg and Ca in these interactions demonstrates that naturally occurring mineral ions from *P. dulce* extract can act as functional dopants, not merely passive stabilizers, thereby contributing to the observed catalytic efficiency.^60^

Overall, the integration of EDS and XPS results provides a comprehensive picture of the nanoparticle interface, revealing how residual phytochemicals and mineral ions together establish a chemically and electronically active surface. These findings underscore the importance of metal-ion coordination and organic capping in modulating both the surface reactivity and functional performance of AuNPs, reinforcing the mechanistic understanding of green-synthesized nanoparticles (Figure 3).

**Figure 3:**
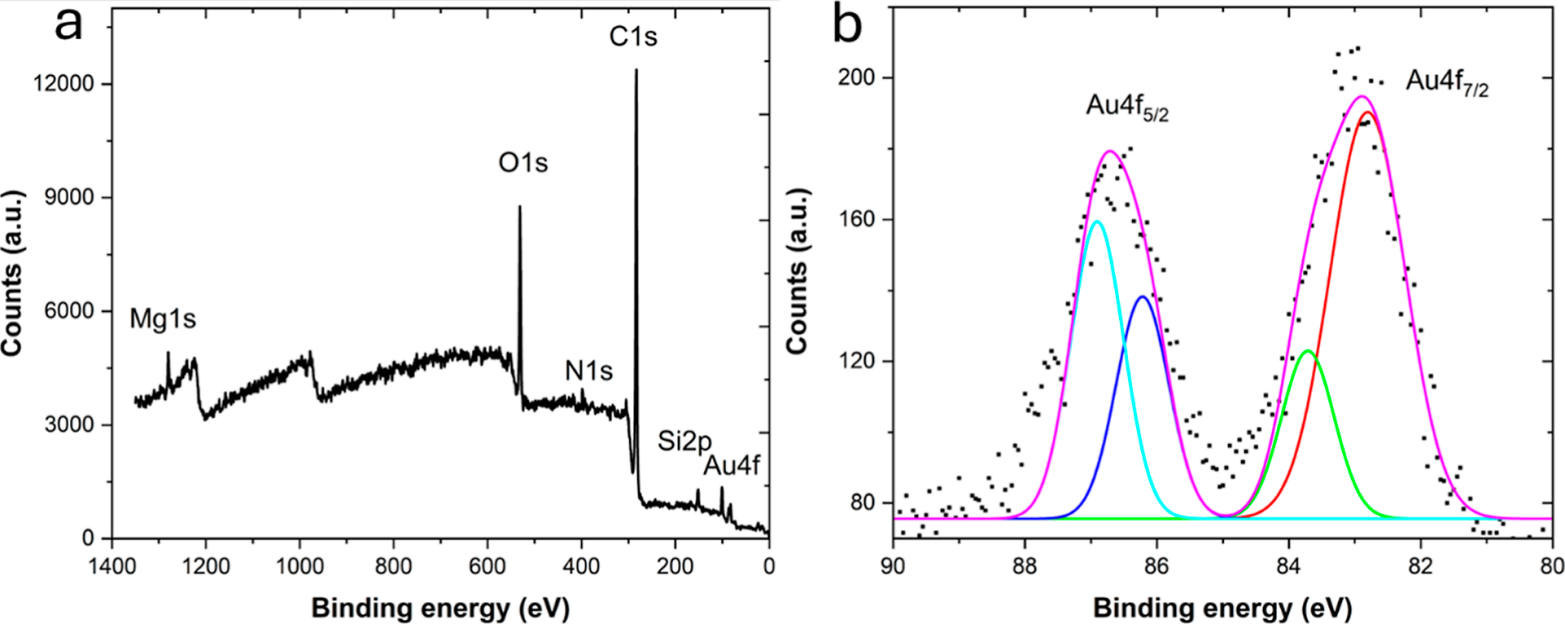
XPS spectrum (a) survey scan and (b) deconvoluted high resolution Au 4f of AuNPs confirming the presence of elemental and chemical states. The Si 2p, C 1s, N 1s, and O 1s peaks are detailed in the Supporting Information.

The mechanistic insights into the formation and functionalization of AuNPs synthesized using *P. dulce* infusion are illustrated in Figure 4a. Initially, the *P. dulce* infusion is enriched with metal ions such as calcium (Ca^2+^), magnesium (Mg^2+^), and potassium (K^+^), which play a pivotal role during nanoparticle synthesis.

**Figure 4:**
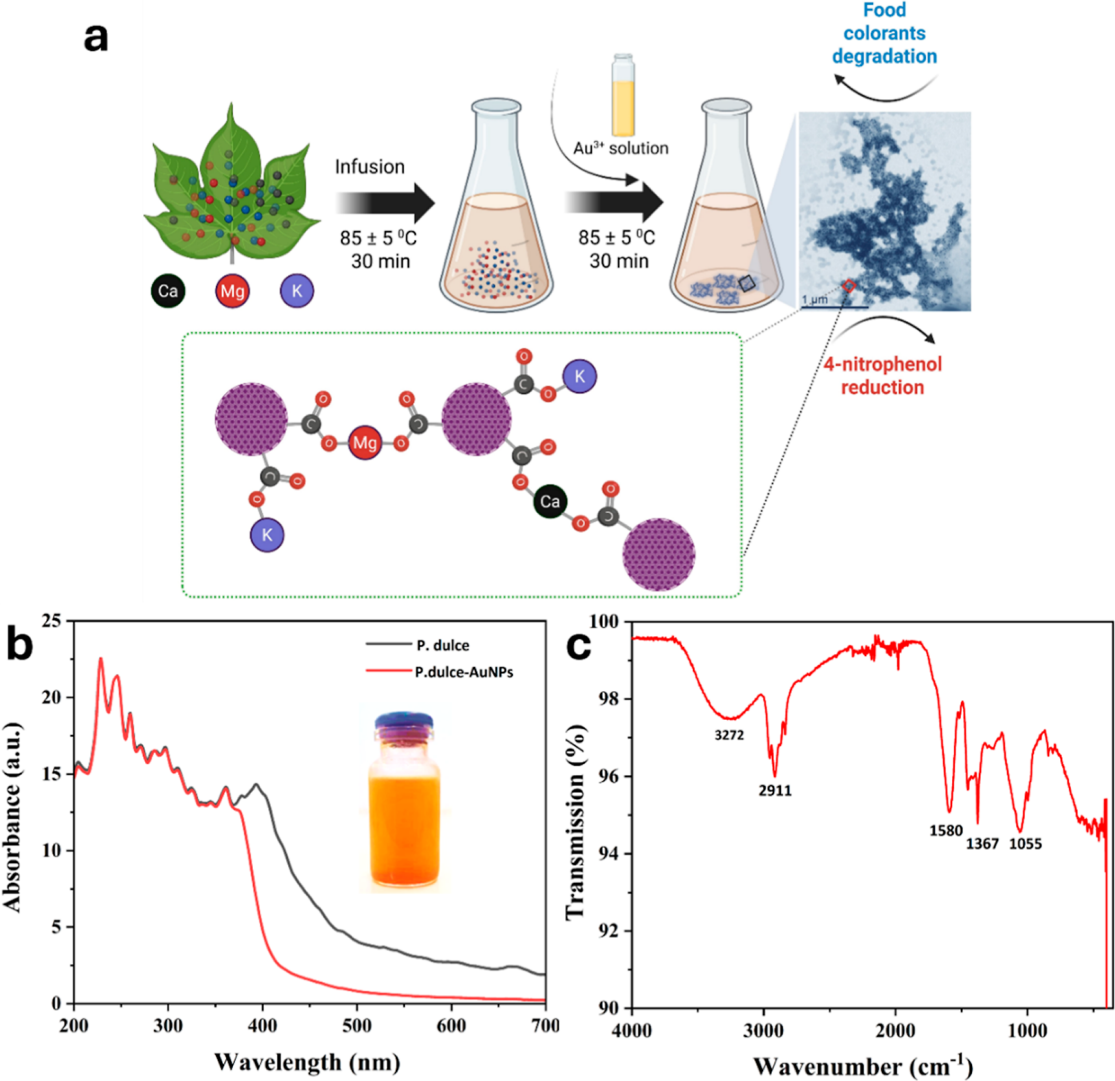
(a) Schematic representation of the metal-ion coordination mechanism involved in the biosynthesis and self-assembly of AuNPs using *P. dulce* infusion, highlighting the role of calcium, magnesium, and potassium ions in nanoparticle surface functionalization and stabilization. (b) UV–visible absorption spectra of *P. dulce* infusion and synthesized AuNPs, exhibiting characteristic peaks related to phytochemicals and nanoparticle optical properties, which illustrate successful nanoparticle formation. (c) FTIR spectra of AuNPs synthesized with *P. dulce* infusion, displaying key absorption bands corresponding to phenolic, carboxylic, and carbohydrate functional groups, confirming their involvement in nanoparticle reduction, stabilization, and surface functionalization.

Upon addition of chloroauric acid, these ions facilitate the formation of metal-ion coordinated, self-assembled AuNPs. The schematic representation highlights coordination interactions between surface functional groups of the nanoparticles—primarily carbonyl (C=O) moieties and metal ions, exemplified by complexes such as C=O–Mg–O–C=O, with analogous coordination involving potassium and calcium ions (Figure S2). This metal-ion coordination network on the AuNP surface underpins both the structural assembly and catalytic functionality of the nanoparticles, which are effectively applied in the reduction of 4-nitrophenol and the degradation of food colorants. Figure 4b presents the UV–Vis absorption spectra of both the *P. dulce* infusion and the synthesized AuNPs.

The infusion displays a pronounced absorption band at ∼393 nm, attributable to phytochemical constituents such as flavonoids and phenolic acids. Upon nanoparticle formation, the spectrum displays a broad feature centered near 361 nm. Notably, it lacks the characteristic surface plasmon resonance (SPR) peak typically observed between 520 and 550 nm for spherical AuNPs, despite the orange coloration of the colloid. However, the reaction was monitored in real-time through UV–visible measurements, and no SPR signal emerged throughout the measurement period. This absence likely reflects a combination of factors, including metal ion-induced self-assembly and the adsorption of “organic molecules”, which are carboxylic groups, on the nanoparticle surfaces. These organic molecules influence the formation of nanoparticle self-assembly, and promote anisotropic morphologies or aggregated structures, leading to broadening, shifting, or suppression (in this case) of the SPR band. The observed spectral profile suggests that secondary metabolites exert a partial stabilization effect, directing particle growth and limiting extensive agglomeration.^61–63^ These combined effects (spectral overlap, dielectric damping, and morphology-driven plasmon perturbation) can broaden, red-shift, or effectively suppress the observable SPR band. The resulting spectral profile therefore reflects a balance between masking by phytochemical absorption and intrinsic nanoparticle properties shaped by the green synthesis conditions.

Complementing these observations, the FTIR spectrum depicted in Figure 4c reveals key functional groups associated with the nanoparticle surface chemistry. The broad absorption band centered at 3272 cm^–1^ corresponds to O–H stretching vibrations, indicative of phenolic hydroxyl groups or adsorbed water molecules. The peak at 2911 cm^–1^ is assigned to C–H stretching vibrations of methyl and methylene groups. The band at 1580 cm^–1^ may be attributed to C=C stretching vibrations within aromatic rings or the asymmetric stretching of carboxylate (−COO^–^) groups, underscoring the presence of phenolic and carboxylic acid functionalities. Additional bands at 1367 cm^–1^ and 1055 cm^–1^ correspond to C–H bending and C–O stretching vibrations, respectively. Collectively, these spectral features confirm the adsorption of organic phytochemicals from *P. dulce* infusion onto the AuNPs surface, highlighting their dual role as reducing and stabilizing agents in the biosynthesis. This biofunctionalization not only influences the physicochemical properties of the nanoparticles but also contributes critically to their catalytic performance.

## 4 Application of 4-NP Reduction

The catalytic activity of AuNPs synthesized via *P. dulce* infusion was evaluated through the reduction of 4-nitrophenol (4-NP) in the presence of sodium borohydride (NaBH_4_). Two different volumes of the AuNP colloidal solution (5 and 10 μL) were tested to assess the influence of nanoparticle concentration on the reaction kinetics and efficiency.

Our results demonstrated that the reduction of 4-NP proceeded with higher apparent rate constants (*K*_app_) when using the lower volume of AuNPs. Specifically, for 5 μL of AuNPs, a *K*_app_ of 0.49 ± 0.18 min^–1^ was obtained, indicating more rapid catalytic activity compared to a *K*_app_ of 0.077 ± 0.003 min^–1^ for 10 μL. Interestingly, the 5 μL AuNP system achieved near-complete reduction within approximately 25 min, whereas the 10 μL system required about 30 min to reach similar conversion levels. Figure 5a illustrates the time-dependent UV–Vis absorbance spectra during 4-NP reduction with AuNPs synthesized from *P. dulce* infusion, showing the gradual disappearance of the 4-nitrophenolate ion peak (∼400 nm) alongside the emergence of peaks corresponding to 4-aminophenol (4-AP), Figure 5b depicts the analogous process for 5 μL of AuNPs, confirming the accelerated reaction kinetics at this lower volume.

**Figure 5:**
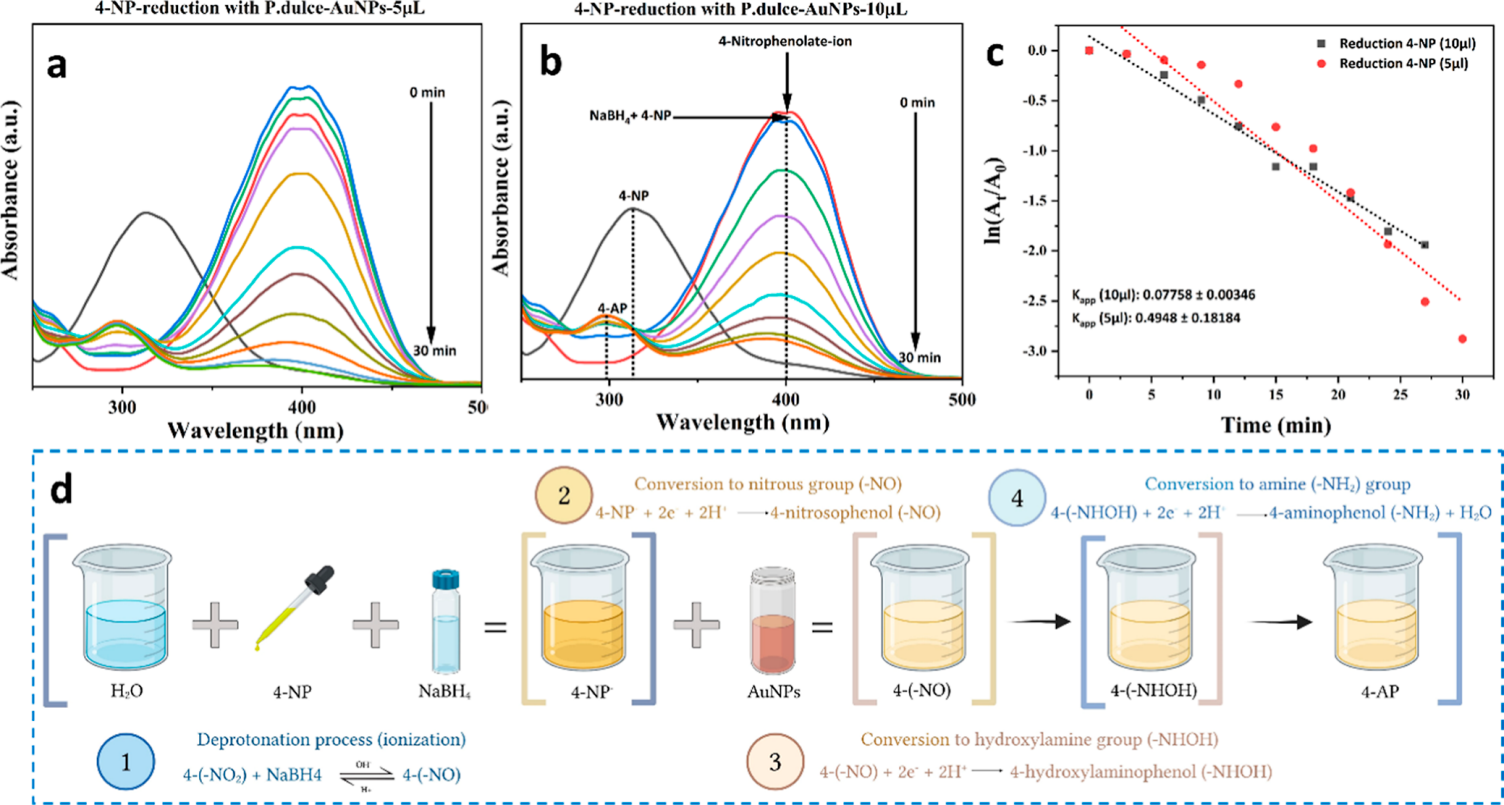
(a) Time-resolved UV–visible absorption spectra of 4-nitrophenol reduction catalyzed by AuNPs synthesized using *P. dulce* infusion, highlighting the decrease of the 4-nitrophenolate ion peak (∼400 nm) and the emergence of 4-aminophenol peaks. (b) Comparison of reduction kinetics using 5 μL of AuNPs, showing accelerated reaction progress relative to higher volumes. (c) Kinetic plots depicting the apparent rate constants (*K*_app_) for 4-NP reduction with 5 and 10 μL of AuNP colloidal solutions. Error bars represent the standard deviation from three independent experiments. (d) Schematic illustration of the proposed multistep reduction mechanism of 4-nitrophenol to 4-aminophenol via intermediates: nitrophenolate ion, nitroso, hydroxylamine, and amine groups.

The kinetic analysis presented in Figure 5c quantitatively compares the reduction rates, further reinforcing the observed trend in catalytic efficiency. The multistep reduction pathway of 4-NP, schematically illustrated in Figure 5d, involves several sequential steps, each influenced by the physicochemical properties of the Au nanoparticles. Initially, 4-NP undergoes deprotonation under alkaline conditions to form the nitrophenolate ion, which exhibits markedly higher adsorption affinity toward the AuNP surface. This adsorption positions the molecule in an orientation that optimizes electronic interactions, facilitating efficient electron transfer from BH_4_^–^. Following adsorption, the nitrophenolate is reduced sequentially to a nitroso intermediate, then to a hydroxylamine derivative, and ultimately to 4-aminophenol. At each stage, the AuNPs function as electron relay centers, stabilizing intermediate reaction and promoting concerted electron and proton transfers, which effectively lowers the activation barriers and accelerates the overall reaction.

The structural characteristics of the AuNPs—including size, shape, and surface morphology—play a crucial role in determining their catalytic performance. Smaller nanoparticles present a higher surface-to-volume ratio, increasing the density of active sites available for substrate and hydride adsorption. Morphological features such as edges, corners, and facets further enhance catalytic activity by providing sites with higher electronic unsaturation and stronger substrate binding. Conversely, partial aggregation or poor dispersion reduces accessible surface area, limiting electron transfer efficiency, as observed in the 10 μL AuNP system.

The bioactive components present in the *P. dulce* extract, including polyphenols, flavonoids, and other phytochemicals, contribute to nanoparticle stabilization by preventing aggregation and maintaining the dispersion of nanoparticles. These molecules may also interact electronically with the Au surface, subtly influencing adsorption orientation and strength for both 4-NP and BH_4_^–^. Such interactions are unique to green-synthesized nanoparticles, where the synergy between biological ligands and nanoparticle surfaces can enhance catalytic efficiency beyond what is achievable with chemically synthesized counterparts.

The higher apparent rate observed with 5 μL of AuNPs, relative to 10 μL, highlights the critical interplay between nanoparticle dispersion, active site accessibility, and surface chemistry. Well-dispersed nanoparticles maximize surface accessibility and maintain optimal electron transfer pathways, whereas higher volumes can promote aggregation, diminishing effective catalytic surface area. These results underscore that in green-synthesized AuNP systems, catalytic performance depends on a balance of size, morphology, dispersion, surface chemistry, and biomolecular capping.

These findings suggest that nanoparticle volume and dispersion critically affect catalytic performance, potentially due to variations in accessible active surface area, particle aggregation state, or surface chemistry. The superior catalytic rate observed with 5 μL AuNPs may be related to enhanced dispersion and active site availability, although further mechanistic studies are necessary to fully elucidate these phenomena.

It is important to remember that the reduction process serves a detoxification role. The primary product, 4-aminophenol (4-AP), is widely used in the pharmaceutical and dye industries and is generally considered less hazardous than 4-nitrophenol.^56,57^ However, several studies have reported that 4-AP may still pose environmental risks and moderate ecotoxicity in aquatic organisms when present at high concentrations.^58^ Therefore, while the catalytic reduction of 4-NP to 4-AP represents a detoxification pathway, the complete environmental safety of the process cannot be assumed without subsequent treatment or degradation of 4-AP. These considerations underscore the importance of integrating catalytic performance with assessments of ecological impact in future applications of green-synthesized nanoparticles.

## 5 Application Degradation of Azo Food Dyes

The catalytic performance of AuNPs synthesized using *P. dulce* leaf infusion was assessed through the reduction of several synthetic food dyes, including Tartrazine (E102), Allura Red AC (E129), Brilliant Blue FCF (E133), and a green formulation composed of a mixture of E102 and E133. These dyes, widely used in the food industry and recognized for their persistence in aquatic environments, represent an important class of organic pollutants. Upon exposure to AuNPs in the presence of sodium borohydride (NaBH_4_), all dye solutions exhibited a marked decrease in coloration within 30 min, indicating effective catalytic degradation. UV–Vis spectroscopic analysis revealed substantial reductions in absorbance intensity alongside spectral narrowing for each dye, confirming the disruption of chromophoric azo structures.

Among the individual dyes, Allura Red AC (E129) exhibited the highest catalytic degradation rate, with an apparent rate constant (*K*_app_) of 0.28061 ± 0.07565 min^–1^ and a removal efficiency of 97.11%, suggesting a strong affinity of this molecule for the nanoparticle surface and efficient electron transfer. Brilliant Blue FCF (E133) followed closely with a *K*_app_ of 0.20753 ± 0.04124 min^–1^ and a removal efficiency of 98.99%. Tartrazine (E102) presented a comparatively slower reaction rate, with a *K*_app_ of 0.12014 ± 0.01639 min^–1^ and 93.81% removal efficiency, likely reflecting differences in molecular structure, charge distribution, and adsorption behavior on the AuNP surface. Furthermore, the green dye system, formulated as a mixture of E102 and E133, achieved a removal efficiency of 97.95%, with a linear regression coefficient (*R* ^2^) of 0.98796 and an apparent rate constant *K*_app_ of 0.19925 ± 0.00898 min^–1^.

This result demonstrates that the catalytic system remains effective even under more complex, multicomponent conditions where competitive adsorption and reduction dynamics typically limit reaction efficiency. Control experiments conducted with sodium borohydride (NaBH_4_) alone, in the absence of AuNPs, showed only minor changes in absorbance after 30 min, confirming that the reducing agent by itself is insufficient to achieve effective dye degradation. This finding underscores the essential role of the biogenic AuNPs in facilitating surface-mediated electron transfer, thereby enabling the breakdown of recalcitrant dye molecules under mild reaction conditions.

The time-dependent UV–visible spectra for each dye Figure 6a–d illustrate the gradual decolorization over time, while the corresponding kinetic plots Figure 6e confirm the pseudo-first-order kinetics of the catalytic degradation process. Collectively, these results demonstrate the potential of AuNPs synthesized from *P. dulce* infusion as efficient and sustainable catalysts for the remediation of dye-contaminated water.

**Figure 6:**
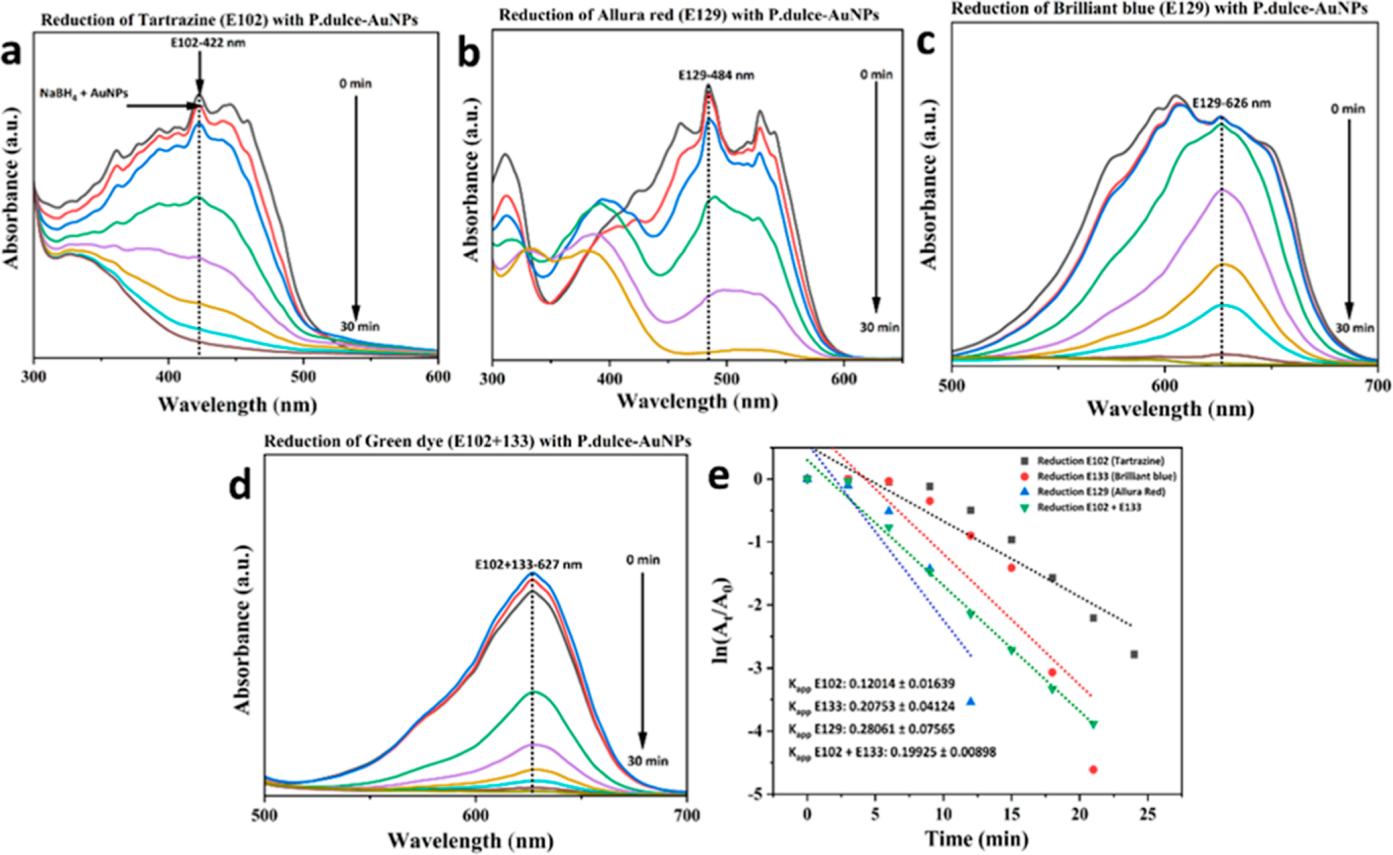
(a) Time-dependent UV–visible absorption spectra showing the catalytic degradation of yellow Tartrazine (E102) using AuNPs synthesized from *P. dulce* infusion. (b) Spectral changes for red dye Allura Red AC (E129). (c) Degradation profile of blue dye Brilliant Blue FCF (E133). (d) Spectra for green dye, a binary mixture of Tartrazine and Brilliant Blue (E102 + E133). (e) Kinetic plots of the pseudo-first-order rate constants (*K*_app_) for the reduction of each dye, confirming the catalytic efficiency of the biogenic AuNPs. Error bars represent the standard deviation from three independent experiments.

The catalytic degradation of azo food dyes mediated by AuNPs synthesized using *P. dulce* infusion involves a series of surface-mediated processes that exemplify the principles of heterogeneous catalysis. Azo dyes such as Tartrazine (E102), Allura Red AC (E129), and Brilliant Blue FCF (E133) (as well as their mixtures) possess extended conjugated systems and azo (−N=N−) groups, which confer their vibrant coloration and chemical persistence.^64–69^

The challenge of degrading these compounds lies in the inherent stability of their chromophoric bonds, requiring efficient catalytic systems to initiate their transformation. Various degradation mechanisms have been reported in the literature for dye removal, depending on the nature of the catalytic or adsorptive material employed.^70,71^ These include bioremediation using bacteria, fungi, or algae; advanced reduction processes such as the Fenton reaction (Fe^2+^/H_2_O_2_); photocatalysis with semiconductors like TiO_2_, ZnO, or g-C_3_N_4_; adsorption on porous materials such as activated carbon, zeolites, metal–organic frameworks (MOFs), or biochars;^72–74^ and, finally, heterogeneous catalytic reduction^75–80^ the mechanism adopted in this work. This latter pathway, often involving noble metal nanoparticles such as Au, Ag, or Pd, is particularly attractive due to its high efficiency, mild reaction conditions, and environmental compatibility.^81–83^

In the present study, the degradation of the tested azo dyes follows a heterogeneous catalytic reduction pathway, catalyzed by AuNPs synthesized with *P. dulce* infusion in the presence of sodium borohydride (NaBH_4_) as a reducing agent. Despite structural variations among the dyes, they share critical functional motifs: the azo bond (−N=N−), which serves as the main chromophore, and sulfonate groups (−SO_3_^–^), which enhance solubility and reactivity in aqueous environments. These common features lead to a similar degradation mechanism across the dye set. The catalytic process begins with system activation. Upon mixing NaBH_4_, the dye solution, and the AuNPs, BH_4_^–^ ions are rapidly generated and act as strong electron donors. Simultaneously, the AuNPs—well-dispersed due to phytochemical stabilization—offer high surface area for catalytic interactions. Dye molecules anchor to the nanoparticle surface through electrostatic interactions between sulfonate groups and metal ions such as Ca^2+^, Mg^2+^, or K^+^ present on the AuNP surface, and via van der Waals interactions with the azo groups. Meanwhile, BH_4_^–^ ions adsorb onto the nanoparticle surface and localize near the dye’s reactive sites. Electron transfer from BH_4_^–^ to the azo groups, facilitated by the AuNPs, reduces the azo bond to a hydrazo intermediate and subsequently to aromatic amines (−NH_2_). This reaction breaks the conjugated π-system, resulting in chromophore deactivation and visible decolorization.

These transformations were monitored and supported by UV–visible spectroscopy, where the characteristic absorbance peaks of each dye decreased significantly during the reaction. Once the chromophores are cleaved, the degradation products—typically aromatic amines—desorb from the nanoparticle surface, regenerating active sites for further reaction cycles. Identified or expected degradation products include sulphanilamide and 4-aminobenzenesulfonic acid for E102; 1-amino-2-naphthol-6-sulfonic acid and sulfonated naphthylamines for E129; and sulfonated benzene and phenylamine derivatives for E133. This stepwise catalytic process is schematically depicted in Figure 7a–d, showing the progressive spectral changes and transformation pathways for each dye.

**Figure 7:**
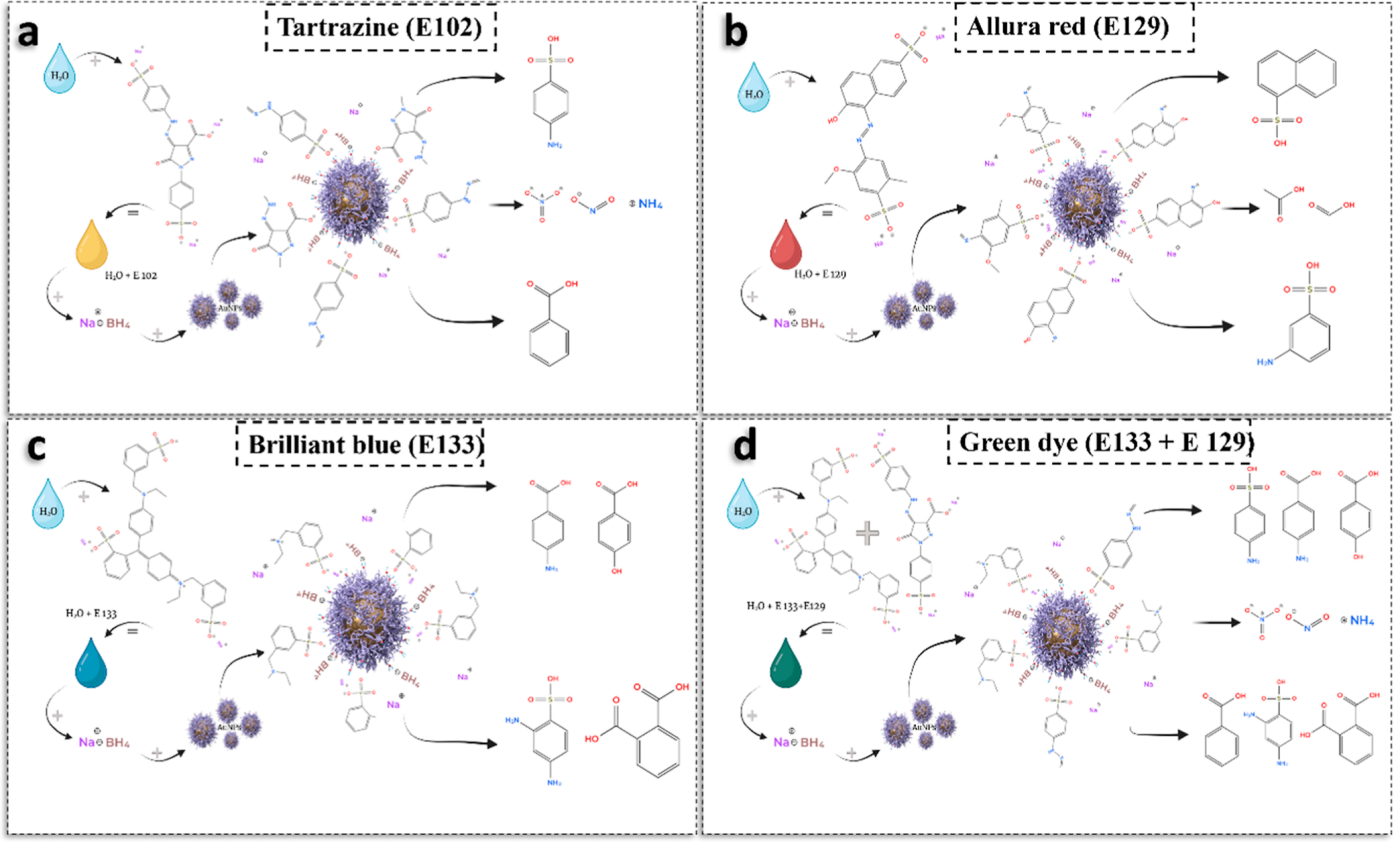
Proposed degradation mechanisms of azo food dyes catalyzed by *P. dulce*-derived AuNPs in the presence of NaBH_4_. (a) Tartrazine (E102), (b) Allura Red AC (E129), (c) Brilliant Blue FCF (E133), and (d) Green dye (mixture of E102 and E133). The scheme illustrates key steps including dye adsorption, azo bond cleavage, and formation of aromatic amine derivatives. The catalytic pathway involves electron transfer from BH_4_^–^ to the azo group, mediated by the nanoparticle surface, with desorption of degradation products completing the catalytic cycle.

The synergistic action of the *P. dulce*-derived phytochemicals and the coordinated metal ions adsorbed on the AuNP surface plays a critical role in facilitating and modulating the catalytic degradation mechanism. To evaluate the relative performance of the green-synthesized AuNPs, their catalytic efficiency was compared with that of other nanomaterials reported in the literature for contaminant removal through catalytic or photocatalytic strategies. Table 2 provides a comprehensive overview of relevant systems, including information on synthesis methods, operative mechanisms, contaminant concentrations, removal efficiencies, *K*_app_, where available. This comparison underscores the effectiveness and competitiveness of the *P. dulce*-based AuNPs within the broader context of sustainable nanotechnology for environmental remediation.

**Table 2:** Performance of Nanomaterials in the Removal of Water Contaminants through Catalytic and Photocatalytic Processes^a^

contaminant	nanomaterial	synthesis method	reduction mechanism	concentration	% removal	*R* ^2^	*K*_app_ (min^–1^)	reference
4-nitrophenol	AgNPs	green synthesis	catalysis	50 mM	99.9	0.995	0.61127	^70^
	AgAu–SiO_2_	green synthesis	catalysis	1 mM	99.9	0.98	0.165	^71^
	Cu@Zn@800	pyrolysis	catalysis	20 mg/L	99.5	—	—	^81^
	AgFe NPs	green synthesis	catalysis	1.4 × 10^–4^ M	78.4	0.94	0.0328 ± 0.0031	^82^
	AuNPs/CQDs	hydrothermal/reduction	catalysis	1 mM	not reported	0.9708	0.1218	^83^
	AuNPs (synthesized via P. dulce infusion)	green synthesis (plant infusion)	catalysis	100 μL	94.37	0.9136	0.49 ± 0.18	this work
tartrazine	75:25 NiFe_2_O_4_/g-C_3_N_4_	coprecipitation + calcination	photocatalysis	50 mg/L	99.65 ± 0.25	—	0.1165	^75^
	NiFe_2_O_4_	coprecipitation + calcination	photocatalysis	50 mg/L	98.08 ± 1.00	0.9912	0.0285	^76^
	BiFeO_3_	not specified	photocatalysis	10 mg/L	95.4	—	0.05	^77^
	g-C_3_N_4_	thermal polycondensation	photocatalysis	50 mg/L	93.76 ± 0.72	0.9915	0.0237	^78^
	Ben@Fe–Cu–Ag	not specified	catalysis	100 mg/L	61.9 ± 1.38	0.2259	0.0018	^84^
	Ag_ZnO NPs	green synthesis	photocatalysis	10 mg/L	15.06	0.998	0.0475	^79^
	TiO_2_/Ni–Co nanowires	hydrothermal	photocatalysis	50 mg/L	not reported	—	—	^80^
	AuNPs (synthesized via P. dulce infusion)	green synthesis (plant infusion)	catalysis	100 μL dye	93.81	0.884	0.12014 ± 0.01639	this work
Allura Red	CuO nanosheets	not specified	photocatalysis	1 mg/10 mL	96.99	—	0.524	^64^
	F–Fe–TiO_2_/SiO_2_	sol–gel	photocatalysis	10 mg/L	96.32	—	—	^65^
	CS-NiSe	precipitation/reduction	photocatalysis	100 mg/L	96.12	—	—	^66^
	porous Ni-doped CuO	wet chemical	photocatalysis	10 mg/L	91.4	—	—	^67^
	CuO NSs	not specified	catalysis	10 mg/L	90.54	—	0.0046	^68^
	S/AgNPs-glu	coprecipitation	catalysis	45 mg/L	72.2	0.9986	0.035	^69^
	AuNPs (synthesized via P. dulce infusion)	green synthesis (plant infusion)	catalysis	100 μL	97.11	0.821	0.28061 ± 0.07565	this work
Brilliant Blue	not specified	not specified	electrocoagulation	5 mg/L	98.97	0.8361	0.0012	^72^
	not specified	not specified	electrocoagulation	0.1 g/50 mL	85.78	0.996	0.0161	^73^
	Not specified	not specified	HPLC-DAD	not specified	not reported	0.991	0.085	^74^
	AuNPs (synthesized via P. dulce infusion)	green synthesis (plant infusion)	catalysis	100 μL dye + 5 μL AuNPs	98.99	0.8084	0.20753 ± 0.04124	this work

a Notes: The reported removal percentages refer to the degradation or transformation of the indicated contaminants under the specified experimental conditions. The symbol “—” (not reported) indicates that the corresponding value was not provided in the referenced publication. The *K*_app_ parameter denotes the apparent rate constant, typically derived from a pseudo-first-order kinetic model. All concentrations are expressed as final contaminant levels in solution, standardized to mg/L or molar equivalents (mM or M). When original units were ambiguous, appropriate conversions were inferred. Building on these findings, the catalytic degradation of azo dyes using AuNPs synthesized with *P. dulce* demonstrates both rapid decolorization and efficient breakdown of chromophoric structures. The resulting aromatic products are simpler and more environmentally manageable than the original dyes. These findings highlight the potential of our green-synthesized nanoparticles as effective agents for removing dye contaminants from aqueous solutions. Importantly, while the degradation products can exhibit ecotoxicity at very high concentrations, under controlled experimental conditions or with proper post-treatment, their environmental impact is minimal. Overall, the results highlight the effectiveness of this catalytic system, which combines high reaction efficiency with consideration of ecological safety in future applications. The classification between catalysis and photocatalysis is based on the mechanism explicitly described in each source. Photocatalytic processes generally involved visible or UV irradiation, although specific wavelengths were not consistently reported. The synthesis method column reflects the primary strategy employed to produce or assemble the nanomaterials; in some cases, the synthesis may involve combined or sequential steps. It is worth noting that the system labeled Green (Tartrazine + Brilliant Blue) achieved a removal efficiency of 97.95%, with a corresponding linear regression coefficient (*R* ^2^) of 0.98796. However, this result is not included in the main table, as it corresponds to a combined dye system rather than the degradation of a single contaminant.

## 6 Conclusions

AuNPs were successfully synthesized using an aqueous infusion of *P. dulce* leaves through a green, cost-effective, and environmentally friendly route. The resulting nanomaterials exhibited a crystalline face-centered cubic (FCC) structure with an average particle size of approximately 10 nm, as confirmed by XRD and HRTEM analyses. The phytochemical constituents present in the infusion, including polyphenols and flavonoids, served as both reducing and capping agents, contributing to the dispersion, and controlled morphology of the nanoparticles. Additionally, coordination with metal cations such as Ca^2+^ and Mg^2+^ adsorbed on the AuNP surface confirmed through XPS study. Spectroscopic and microscopic characterizations revealed that these biomolecules not only stabilized the AuNPs but also modulated their self-assembly and surface functionality.

The synthesized AuNPs displayed excellent catalytic performance in the reduction of 4-NP and the degradation of various azo food dyes (Tartrazine, Allura Red AC, Brilliant Blue FCF, and their mixtures) in the presence of NaBH_4_. The catalytic activity surpassed that of the reducing agent alone, demonstrating the crucial role of the biogenic nanoparticles in facilitating electron transfer and chromophore degradation. The observed kinetics support the potential of these materials as effective catalysts for the remediation of molecular pollutants in aqueous systems. While the resulting degradation products are generally simpler and more environmentally manageable than the parent dyes, their environmental impact should still be considered, particularly at higher concentrations, underscoring the importance of controlled conditions or post-treatment steps.

Overall, this study highlights the potential of *P. dulce*-mediated green synthesis as a sustainable approach for fabricating biofunctionalized AuNPs with broad applicability in catalysis and environmental detoxification. Furthermore, future work will explore strategies to enable the reusability and recovery of these nanocatalysts, evaluate the toxicity of degradation byproducts, and extend the study to real wastewater samples to confirm environmental safety and practical applicability.

## Supplementary Material



## Notes

The average crystallite size of the biosynthesized gold nanoparticles was determined using the Scherrer equation, data processing, tools used to calculate the average interplanar distance from high-resolution transmission electron microscopy micrographs, and high-resolution XPS analysis of C 1s. O 1s, N 1s, and Si 2p are presented (PDF)
