# Supplementary material for: Metal-Ion Coordination Self-Assembled Green Synthesized Gold Nanoparticles for 4‑Nitrophenol Reduction and Food Colorants Degradation
Source: ACS Omega. 2025 Sep 22;10(39):46025–39. doi: 10.1021/acsomega.5c06971 (PMC12509122; doi:10.1021/acsomega.5c06971)
Supplement: Supplementary file 1 [file ao5c06971_si_001.pdf]

Supporting Information:

Metal-ion Coordination Self-Assembled Green Synthesized Gold Nanoparticles For 4-Nitrophenol Reduction and Food Colorants Degradation

*Josue Abraham Lara Zavala, Edgar Ivan Ocampo Lopez, Mora Fonseca Monica Cecilia, Helena Cortés Reyes, Eduardo Silva Beltrán, Naveen Kumar Reddy Bogireddy**

Instituto de Ciencias Físicas, National Autonomous University of Mexico (UNAM), Mexico C.P. 04510, Mexico

**Corresponding author email: naveen@icf.unam.mx*

Crystallite size determination by XRD

The average crystallite size of the biosynthesized gold nanoparticles was determined using the **Scherrer equation**:

$$D = \frac{\kappa * \lambda}{\beta * cos\theta} \qquad (1)$$

with *K*= (~0.9 for roughly spherical particles), λ=0.15406 nm, β in radians and θ in radians.

where:

- *D*: is the crystallite size (nm)
- *K*: is the shape factor (~0.9 for roughly spherical particles)
- λ: 0.15406 nm is the X-ray wavelength (Cu-Kα)
- β: is the full width at half maximum (FWHM) in radians
- θ: is the Bragg angle of the diffraction peak in radians

The XRD pattern showed four characteristic peaks corresponding to the (111), (200), (220), and (311) planes of face-centered cubic (FCC) gold at the following 2θ angles:

Plane	2θ (°)	θ (°)	θ (rad)	β (°)	β (rad)
(111)	38.33	19.165	0.33449	0.41	0.007155
(200)	44.44	22.22	0.3878	0.962	0.0167
(220)	64.89	32.445	0.56627	0.63	0.01099
(311)	77.71	38.855	0.67814	0.7492	0.01307

Substituting these values into Equation (1) gives the following crystallite sizes:

$$D = \frac{(0.9 * 0.15406)}{(0.007155 * \cos(0.33449))} = 19.3789\, nm$$

$$D = \frac{(0.9 * 0.15406)}{(0.0167 * \cos(0.3878))} = 8.3028\, nm$$

$$D = \frac{(0.9 * 0.15406)}{(0.01099 * \cos(0.56627))} = 12.6169\, nm$$

$$D = \frac{(0.9 * 0.15406)}{(0.01307 * \cos(0.67814))} = 10.6093\, nm$$

Average crystallite size: 12.73 nm

This value agrees well with the HRTEM results (**11.63 ± 0.43 nm**), confirming the consistency of the particle size determination.

Note: Minor differences between XRD and TEM measurements are expected, as XRD estimates the size of coherent crystalline domains (which may include aggregates or polycrystalline regions), whereas TEM directly measures individual nanoparticles

Supplementary Information: Determination of Interplanar Spacing in Gold Nanoparticles

Analysis Overview

An analysis of three high-resolution transmission electron microscopy (HRTEM) micrographs was conducted to determine the average interplanar spacing of the synthesized nanoparticles. Using digital image processing and Fast Fourier Transform (FFT), crystalline regions were identified, and the distances between atomic planes were calculated. The results provide insight into the structural characteristics of the nanoparticles.

Methodology

Selection of Analysis Regions
Regions with clearly visible lattice fringes were selected in each micrograph. These fringes correspond to atomic planes in well-ordered nanoparticles.

Digital Processing

- Application of contrast enhancement and noise reduction filters.
- Fast Fourier Transform (FFT) to determine the periodicity of the lattice fringes.
- Extraction of intensity profiles to measure the distance between consecutive maxima.

Tools Used

- Image analysis software: ImageJ with FFT plugin.
- Calibration based on the 10 nm scale provided in each micrograph.

Results

- **Micrograph 1:** average interplanar spacing: 0.34 nm; standard deviation: ±0.02 nm
- **Micrograph 2:** average interplanar spacing: 0.28 nm; standard deviation: ±0.01 nm
- **Micrograph 3:** average interplanar spacing: 0.31 nm; standard deviation: ±0.02 nm

The measured interplanar spacings are consistent with typical crystalline structures of metallic materials such as gold. Variations among the micrographs can be attributed to differences in

crystallographic orientation. Overall, the results reflect well-defined crystalline structures, indicative of controlled synthesis and potential for functional applications.

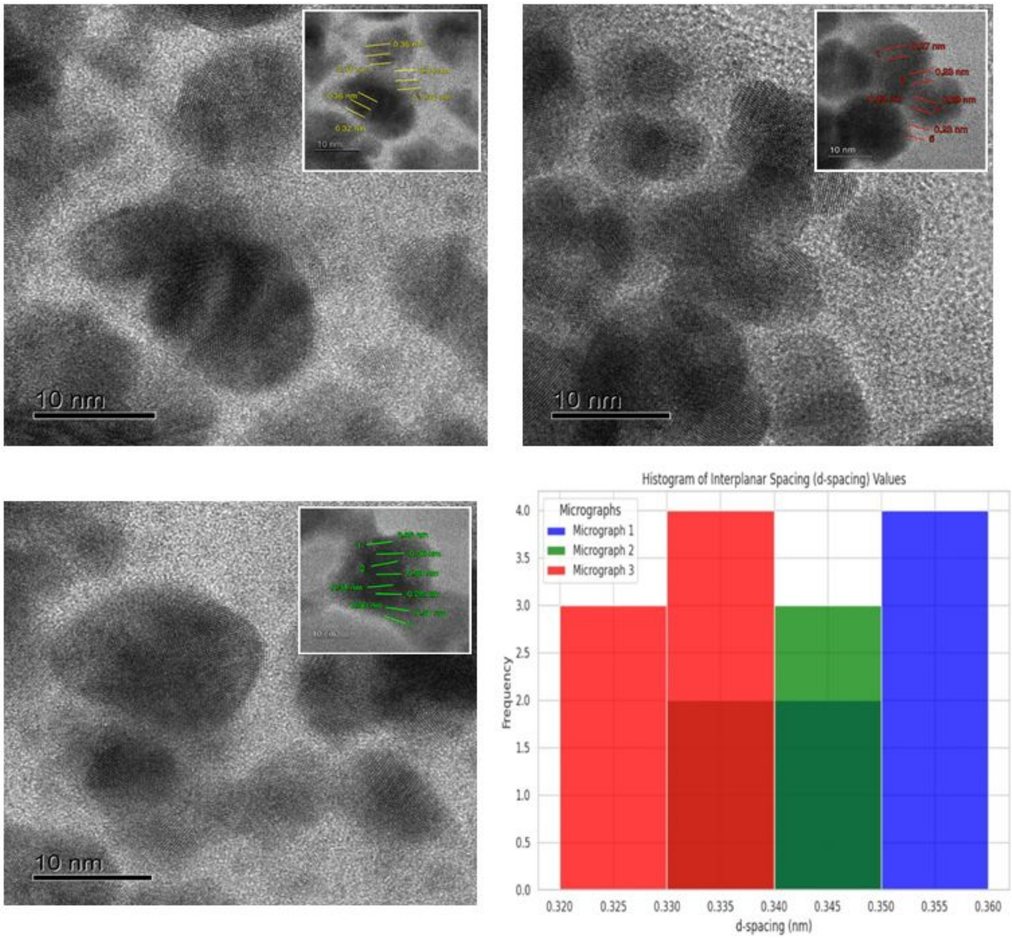

Figure S1. An analysis of three high-resolution transmission electron microscopy (HRTEM) micrographs was conducted to determine the average interplanar spacing of the synthesized nanoparticles. Using digital image processing and Fast Fourier Transform (FFT), crystalline regions were identified, and the distances between atomic planes were calculated. The results provide insight into the structural characteristics of the nanoparticles.

Figure Indicators

- Parallel lines highlighting the visible lattice planes.
- Indicators of interplanar distances (in nanometers).
- Reference scale based on the 10 nm scale bar.

A histogram of the interplanar spacing values from the three micrographs is included, along with the size distribution frequency (in nanometers) for each micrograph. The overall average interplanar

spacing is 0.31 nm ± 0.02 nm. The slight differences may be attributed to the aggregation of nanoparticles observed in the micrographs.

XPS Analysis of Mg^{2+} and Ca^{2+} Coordination on AuNPs

To provide direct evidence of Mg^{2+} and Ca^{2+} coordination on the surface of gold nanoparticles (AuNPs), X-ray photoelectron spectroscopy (XPS) analyses were performed. The survey scans confirm the presence of Mg and Ca on the nanoparticle surface. Deconvolution of the Au 4f peak reveals a shift from 83.9 eV, characteristic of bare Au, to 82.8 eV, indicative of electron transfer between AuNPs and Mg, consistent with coordination interactions. Similarly, the Ca 2p spectra show binding energies corresponding to the presence of calcium ions coordinated to surface functional groups.

These XPS results corroborate the TEM-EDS observations and provide strong evidence for the association of Mg^{2+} and Ca^{2+} with the AuNPs. Representative spectra and additional deconvolutions are presented in Figure 2.

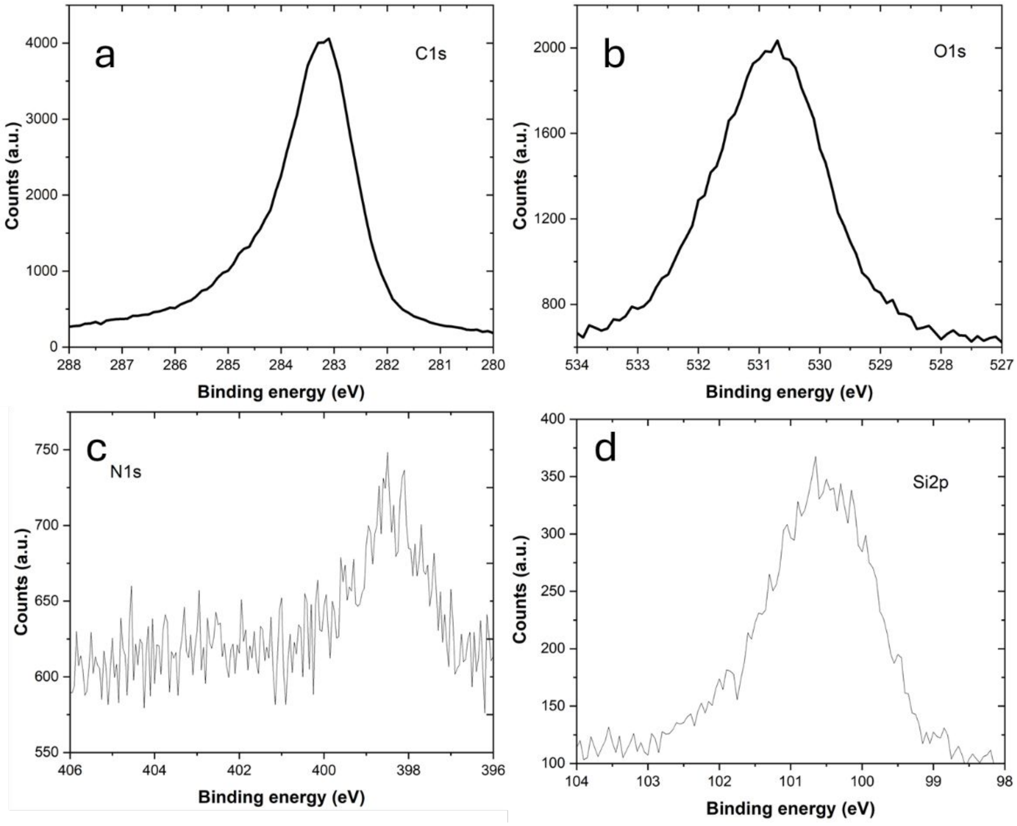

Figure S2. High resolution XPS analysis of (a) C1s. (b) O1s, (c) N1s and (d) Si2p.
